# Setting research priorities around the impact of COVID-19 control measures on people with dementia and caregivers living at home: A 14-country perspective

**DOI:** 10.1177/13872877261451163

**Published:** 2026-05-30

**Authors:** Geeske Peeters, Rachel L. Fitzpatrick, Tatyana Mollayeva, Yaohua Chen, Elisa de Paula França Resende, Tomas Leon, Lina M. Zapata-Restrepo, Kuripacha Tituaña, Magda Tsolaki, Faheem Arshad, Prekshya Thapa, Temitope Farombia, Belen Custodio, Khanyo Ntokozo Ngcobo, Roger O'Sullivan, Iracema Leroi, on behalf of the STRAP consortium

**Affiliations:** 1 Radboudumc Alzheimer Centre, 6034 Radboud University Medical Centre, Nijmegen, The Netherlands; 2 Global Brain Health Institute, University of California San Francisco, Trinity College Dublin, San Francisco, CA, USA; 3 School of Medicine, 8809 Trinity College Dublin, Dublin, Ireland; 4 University Health Network, 7938 University of Toronto, Toronto, Canada; 5 Department of Geriatrics, 27023 University of Lille, CHU Lille, U1172, Lille, France; 6 Faculdade de Medicina and Hospital das Clínicas, 28114 Universidade Federal de Minas Gerais, Belo Horizonte, Brazil; 7 476601 Hospital de Salvador, Salvador, Chile; 8 67597 Fundacion Valle del Lili, Cali, Colombia; 9 185017 Pontificia Universidad Católica del Ecuador-Ibarra, Ibarra, Ecuador; 10 Greek Association of Alzheimer Disease and Related Disorders, Thessaloniki, Greece; 11 29148 National Institute of Mental Health and Neurosciences, Bangalore, India; 12 58594 B.P. Koirala Institute of Health Sciences, Dharan, Nepal; 13 107962 University College Hospital, Ibandan, Nigeria; 14 Instituto Peruano de Neurociencias, Lima, Peru; 15 56394 University of Kwazulu Natal, Durban, South Africa; 16 Ulster University, Coleraine, Northern Ireland; 17 9416 Institute of Public Health, Belfast, Northern Ireland; 18 HRB-CTN Dementia Trials Ireland, Ireland

**Keywords:** Alzheimer's disease, caregivers, community health planning, health policy, health service accessibility, health system resilience, pandemics, research agenda, stakeholder participation

## Abstract

**Background:**

The global impact of COVID-19 restrictions on people with dementia (PWD) living at home and their informal caregivers has been described extensively. However, adoption of this knowledge into policy and practice is limited because of a lack of coordinated, inclusive, and regionally sensitive prioritization.

**Objective:**

To establish key regional research priorities for Europe and the Global South.

**Methods:**

Following consensus-based prioritization methods, we applied a three-step approach: 1) a systematic literature review to derive a list of topics describing how PWD and caregivers were impacted by the COVID-19 restrictions; 2) an online survey distributed to PWD, caregivers and health care professionals (HCP) across 14 countries asking respondents to select the 10 most important topics; and 3) an iterative consultation process with stakeholders from each country to translate the top-ranked topics into a research agenda.

**Results:**

We identified 51 quantitative and 18 qualitative relevant publications, from which we derived 38 topics. 29 PWD, 110 caregivers and 117 HCP across 14 countries prioritized these topics, which largely overlapped across stakeholder groups and countries. The top ranked priorities cluster into four themes: daily routine and physical function, mental health, disease progression, and access to care. The consultancy process with stakeholders resulted in three lines of research to address these themes: understanding mechanisms, designing and improving education, and information access.

**Conclusions:**

This research agenda offers a roadmap to guide future research and policy aimed at strengthening support for PWD and their caregivers in times of public health crises.

## Introduction

The COVID-19 control measures, such as quarantine, social distancing, and reallocating resources, significantly affected people with dementia (PWD) living at home and their informal caregivers.^1,2^ The closure of clinics and support centers, and prolonged periods of cohabitation, led to negative impacts on their well-being.^3,4^ Furthermore, many worried about a more rapid decline in cognitive, behavioral and physical functioning.^5–7^ Both PWD and their caregivers often experienced a deterioration in mental health, as well as increased loneliness, care burden, and stress.^8,9^

During the peak of the pandemic, national and local governments had to swiftly navigate through uncertainties surrounding the virus's behavior, vaccine development timelines, available intensive care unit beds and care staff, the effectiveness of control measures and impact of those measures on other sectors.^10^ With control measures impacting various societal sectors such as healthcare, education, and the economy, policymakers faced challenges in evaluating their consequences across all sectors. They prioritized suppressing the virus's spread over other needs. However, in hindsight, it is evident that these measures also had harmful consequences, especially for PWD and their caregivers. Setting priorities for health research becomes crucial to understanding the impact of COVID-19, particularly for people disproportionately affected by the control measures and those in resource-sparse communities.

This paper describes a mixed-methods approach to: 1) synthesize and prioritize evidence gaps related to the impact of COVID-19 control measures on community-dwelling PWD and their caregivers in Europe and the Global South; and 2) and provide region-specific recommendations for a research agenda that could inform improved care for PWD living at home and their caregivers, both in the context of COVID-19 and in future pandemics or crises. Leveraging our global network of equity for brain health leaders with transdisciplinary expertise, we aimed to ensure that the research priorities were evidence-based and grounded in stakeholder input, to generate the knowledge policymakers need to make better-informed, equitable decisions in future health emergencies.

## Methods

### Ethics

The protocol was approved by the research ethics boards (REBs) at Trinity College Dublin (REB #2023) and the University of Toronto (REB # 44-804). In addition, each consortium partner obtained additional ethical approval within their respective countries (Supplemental Table 1). Some countries considered the priority setting survey a consultancy process rather than research and therefore did not require ethics approval.

### Study design

To develop the research agenda, we followed a three-step, mixed-methods process:

1. A systematic literature review was conducted to identify and categorize consequences of COVID-19 control measures experienced by PWD living at home and their caregivers. This informed a preliminary list of research topics.
2. A priority setting survey was administered to PWD, their caregivers, and HCP across 14 countries to identify the 10 most important topics, allowing us to understand the top priorities for each stakeholder group and country.
3. An iterative consultation was conducted with stakeholders from the participating countries, to refine and contextualize the survey results. This process translated the selected priorities into an actionable research agenda.

*Step 1: Literature review.* The systematic review process is described in detail elsewhere^11^; here we provide a summary relevant to the development of the research agenda. Using search terms (including synonyms) for “dementia”, “mental health”, “COVID-19”, and “caregivers”, we searched Medline, PsycINFO, EMBASE, Web of Science, CINAHL, Latin American and Caribbean Health Literature (Lilacs), Scientific Electronic Library online (Scielo), and EM Premium, covering the period from March 2020 to July 2022, without country or language restrictions. We also searched grey literature using Google Scholar (the first 10 pages), and an inventory of existing national surveys and reports in each participant's country for items published between the start of the pandemic through the end of July 2022. We cross-checked the references list of all included studies.

We included studies that reported results from original quantitative or qualitative studies examining the consequences of COVID-19 control measures on the lives of PWD and/or their caregivers. A team of researchers independently extracted data from all papers so that each paper was extracted by two researchers and a third researcher checked any inconsistencies. Data extraction involved descriptives of each paper and any quantitative or qualitative results that reflect consequences of COVID-19 control measures on disease progression of the PWD, carer burden perceived by the care partner, and physical health, mental health (including loneliness) and access to care/services of both the PWD and the care partner.

From the extracted data, two researchers (YC, RF) independently derived a list of topics that formed the input for the priority setting survey. Prior experience with priority setting surveys learnt that respondents can cope with a maximum of approximately 40 topics. Therefore, five researchers (RF, YC, FF, TM, GP) condensed the long list of topics identified from the review into a list of a maximum of 40 topics by merging topics that covered the same theme through an iterative process until consensus was reached.

*Step 2: Priority setting process.* Based on the short list of topics derived from the literature review, we developed an online survey to assess which topics had the greatest impact on PWD and their caregivers. The survey targeted three stakeholder groups: PWD, caregivers, and HCPs. We developed an initial survey version in English and subsequently refined it based on the iterative feedback process involving stakeholders, including representatives of patient and public involvement (PPI). To ensure accessibility and cross-cultural relevance, the final version of the survey was translated (using DeepL and checked by native speakers) into the languages of participating countries, including Greek, Dutch, Spanish, Portuguese, and other languages.

We aimed to recruit 20 respondents per country to establish equal representation across all countries, ideally a mix of PWD, caregivers, and HCPs. In some countries, ethical restrictions or practical circumstances did not allow us to recruit PWD. We distributed the survey in 14 countries, five of which were across Europe (France, Greece, Ireland, Netherlands, United Kingdom) and nine in the Global South (Brazil, Chile, Colombia, Ecuador, India, Nepal, Nigeria, Peru and South-Africa).

Respondents were asked to review the list of topics and select a maximum of 10 topics that had the greatest impact during the COVID-19 pandemic on PWD and caregivers, and that they felt where research was needed. We also asked respondents to list any additional topics they felt were missing from the list. Finally, we asked them to choose their top three priorities from their previously selected and newly added topics.

Topics were ranked by assigning one point each time a respondent included a topic in their top 10 list, and an additional point if the topic also appeared in their top three list. For each stakeholder group, we then selected the topics with the highest total rank to create priority lists of approximately 10 topics for each stakeholder group. This was done for each of the 14 countries separately (country-specific lists) and for all countries combined (generic list).

*Step 3: Translating priorities into a research agenda.* RF, IL, YC, TM and GP led the translation process by qualitatively analyzing the priority lists across countries and stakeholder groups. Given the large overlap in lists across countries, we decided to initially develop the research agenda based on the generic list. Topics were grouped into overarching themes. Per theme, we discussed the type of research needed to inform knowledge and policy. This process resulted in a draft research agenda, which was then shared with the STRAP consortium for feedback. Through several iterative rounds of consultation (via video calls and email), we refined the agenda until we reached consensus. We then compared each country-specific priority list with the generic list and research agenda. In consultation with the partners of that country, we determined whether the final research agenda required any adaptations or additions to reflect the socio-cultural context, including the structure of the healthcare system, ethical guidelines and approval processes, cultural norms, values, and caregiving practices.

## Results

### Step 1: Literature review

In total, 69 papers (51 quantitative, 18 qualitative) were included describing data of over 260,000 PWD and caregivers. From the extracted data, we derived a list of 72 topics covering the following themes: physical health/daily functioning, cognition, mental health, behavioral problems, wellbeing, access to health services, health and wellbeing of the care partner, and care burden. This list was condensed to 38 topics by merging overlapping topics (Supplemental Table 2).

### Step 2: Priority setting process

In total, 256 people completed the survey across 14 countries: 29 PWD, 110 caregivers, and 117 HCP (Table 1). PWD who responded to the survey were predominantly female and 55 years of age or older (Table 2). Caregivers who responded to the survey were predominantly female and 55 years of age or older (Table 3). HCPs who responded were also predominantly female across all countries (age not asked, Table 4).

**Table 1.** Number of respondents per stakeholder group per country.

Country	Total	People with dementia	Care givers	Care professionals
Brazil	18	2	12	4
Chile	17	1	15	1
Colombia	6	0	0	6
Ecuador	25	0	4	21
France	9	0	3	6
Greece	25	0	15	10
India	12	0	9	3
Ireland	20	4	9	7
Nepal	12	0	4	8
Netherlands	17	0	7	10
Nigeria	24	8	6	10
Peru	20	7	7	6
South Africa	16	0	8	8
UK	16	6	1	9
Unknown/non-participating country	19	1	10	7
Total	256	29	110	117

**Table 2.** Characteristics of people with dementia who responded to the survey.

	Brazil	Chile	Colombia	Ecuador	France	Greece	India	Ireland	Nepal	Netherlands	Nigeria	Peru	South Africa	UK
N	2	1	0	0	0	0	0	4	0	0	8	7	0	6
Age (%)														
<45 years	0	0	n/a	n/a	n/a	n/a	n/a	0	n/a	n/a	0	0	n/a	0
45–54 years	0	0	n/a	n/a	n/a	n/a	n/a	0	n/a	n/a	0	0	n/a	16.7
55–64 years	0	0	n/a	n/a	n/a	n/a	n/a	100	n/a	n/a	0	14.3	n/a	50.0
65–74 years	100	0	n/a	n/a	n/a	n/a	n/a	0	n/a	n/a	62.5	42.9	n/a	16.7
75 + years	0	100	n/a	n/a	n/a	n/a	n/a	0	n/a	n/a	37.5	42.9	n/a	16.7
Gender (%)														
Women	50.0	100	n/a	n/a	n/a	n/a	n/a	25.0	n/a	n/a	25.0	42.9	n/a	16.7
Men	50.0	0	n/a	n/a	n/a	n/a	n/a	50.0	n/a	n/a	62.5	57.1	n/a	66.7
Prefer not to say Not answered	0 0	0 0	n/a n/a	n/a n/a	n/a n/a	n/a n/a	n/a n/a	25.0 0	n/a n/a	n/a n/a	12.5 0	0 0	n/a n/a	0 16.7
Managing on income (%)														
Very difficult	0	0	n/a	n/a	n/a	n/a	n/a	25.0	n/a	n/a	0	14.3	n/a	66.7
Somewhat difficult	0	0	n/a	n/a	n/a	n/a	n/a	25.0	n/a	n/a	37.5	57.1	n/a	0
Not easy/not difficult	0	100	n/a	n/a	n/a	n/a	n/a	25.0	n/a	n/a	0	0	n/a	16.7
Somewhat easy	0	0	n/a	n/a	n/a	n/a	n/a	0	n/a	n/a	25.0	28.6	n/a	0
Very easy Not answered	100 0	0 0	n/a n/a	n/a n/a	n/a n/a	n/a n/a	n/a n/a	25.0 0	n/a n/a	n/a n/a	25.0 12.5	0 0	n/a n/a	0 16.7
Type of dementia (%)														
Alzheimer's disease	100	100	n/a	n/a	n/a	n/a	n/a	0	n/a	n/a	87.5	85.7	n/a	66.7
Vascular dementia	0	0	n/a	n/a	n/a	n/a	n/a	0	n/a	n/a	12.5	0	n/a	0
Lewy body dementias	0	0	n/a	n/a	n/a	n/a	n/a	50.0	n/a	n/a	0	0	n/a	0
FTD	0	0	n/a	n/a	n/a	n/a	n/a	0	n/a	n/a	0	14.3	n/a	16.7
Other/unknown Prefer not to say	0 0	0 0	n/a n/a	n/a n/a	n/a n/a	n/a n/a	n/a n/a	25.0 25.0	n/a n/a	n/a n/a	0 0	0 0	n/a n/a	16.7 0

AD: Alzheimer's disease; FTD: frontotemporal dementia; n/a: not applicable as no people with dementia responded in this country.

**Table 3.** Characteristics of caregivers who responded to the survey.

	Brazil	Chile	Colombia	Ecuador	France	Greece	India	Ireland	Nepal	Netherlands	Nigeria	Peru	South Africa	UK
N	12	15	0	4	3	15	9	9	4	7	6	7	8	1
Age (%)														
<45 years	0	6.7	n/a	100	0	20.0	55.6	0	25.0	0	0	0	12.5	0
45–54 years	16.7	53.3	n/a	0	0	33.3	11.1	11.1	25.0	28.6	0	14.3	12.5	100
55–64 years	41.7	13.3	n/a	0	0	33.3	11.1	55.6	25.0	57.1	0	42.9	37.5	0
65–74 years	33.3	13.3	n/a	0	66.7	0	22.2	11.1	25.0	14.3	0	28.6	25.0	0
75 + years Not specified	0 8.3	13.3 0	n/a n/a	0 0	33.3 0	0 13.3	0 0	0 22.2	0 0	0 0	0 100	0 14.3	12.5 0	0 0
Gender (%)														
Women	83.3	86.7	n/a	75.0	66.7	66.7	44.4	100	75.0	85.7	33.3	71.4	87.5	0
Man	16.7	13.3	n/a	25.0	33.3	20.0	55.6	0	25.0	14.3	50.0	28.6	12.5	100
Prefer not to say Not answered	0 0	0 0	n/a n/a	0 0	0 0	13.3 0	0 0	0 0	0 0	0 0	0 16.7	0 0	0 0	0 0
Managing on income (%)														
Very difficult	16.7	58.3	n/a	25.0	0	20.0	11.1	22.2	0	0	16.7	0	37.5	0
Somewhat difficult	33.3	33.3	n/a	75.0	0	33.3	33.3	33.3	0	0	0	71.4	37.5	0
Not easy/not difficult	16.7	8.3	n/a	0	33.3	20.0	11.1	33.3	75.0	42.9	50.0	0	25.0	100
Somewhat easy	16.7	0	n/a	0	0	6.7	22.2	11.1	25.0	57.1	0	14.3	0	0
Very easy Not answered	16.7 0	0 0	n/a n/a	0 0	66.7 0	20.0 0	22.2 0	0 0	0 0	0 0	16.7 16.7	14.3 0	0 0	0 0
Type of dementia (%)														
Alzheimer's disease	91.7	46.7	n/a	25.0	33.3	20	55.6	25	75.0	0	0	71.4	37.5	0
Vascular dementia	0	13.3	n/a	0	33.3	13.3	0	25	0	33.3	0	0	12.5	100
Lewy body dementias	0	6.7	n/a	0	0	0	0	12.5	0	16.7	0	0	0	0
FTD	0	6.7	n/a	0	0	6.7	44.4	0	0	33.3	0	28.6	0	0
Other/unknown Not specified	0 8.3	20 6.7	n/a n/a	75.0 0	0 33.3	46.7 13.3	0 0	12.5 25	25.0 0	16.7 0	0 100	0 0	50.0 0	0 0

AD: Alzheimer disease; FTD: frontotemporal dementia; n/a: not applicable as no caregivers responded in this country.

**Table 4.** Characteristics of care professionals who responded to the survey.

	Brazil	Chile	Colombia	Ecuador	France	Greece	India	Ireland	Nepal	Netherlands	Nigeria	Peru	South Africa	UK
N	4	1	6	21	6	10	3	7	8	10	10	6	8	9
Gender (%)														
Women	100	100	83.3	66.7	100	60.0	33.3	42.9	75.0	100	40.0	83.3	87.5	77.8
Men	0	0	16.7	23.8	0	40.0	33.3	28.6	25.0	0	60.0	16.7	12.5	22.2
Prefer not to say	0	0	0	9.5	0	0	33.3	28.6	0	0	0	0	0	0
Managing on income (%)														
Very difficult	0	0	0	33.3	0	20.0	0	14.3	75.0	0	20.0	0	25.0	0
Somewhat difficult	0	100	16.7	28.6	0	30.0	0	14.3	0	0	0	33.3	12.5	0
Not easy/not difficult	50	0	50	28.6	0	40.0	33.3	0	12.5	20	60.0	33.3	25.0	75.0
Somewhat easy	0	0	0	4.8	50.0	0	66.7	42.9	12.5	30	20.0	33.3	25.0	25.0
Very easy	50	0	33.3	4.8	50.0	10.0	0	28.6	0	30	0	0	12.5	0
Not answered	0	0	0	0	0	0	0	0	0	20	0	0	0	0

n/a: not applicable as no care professionals responded in this country.

**Figure 1.**
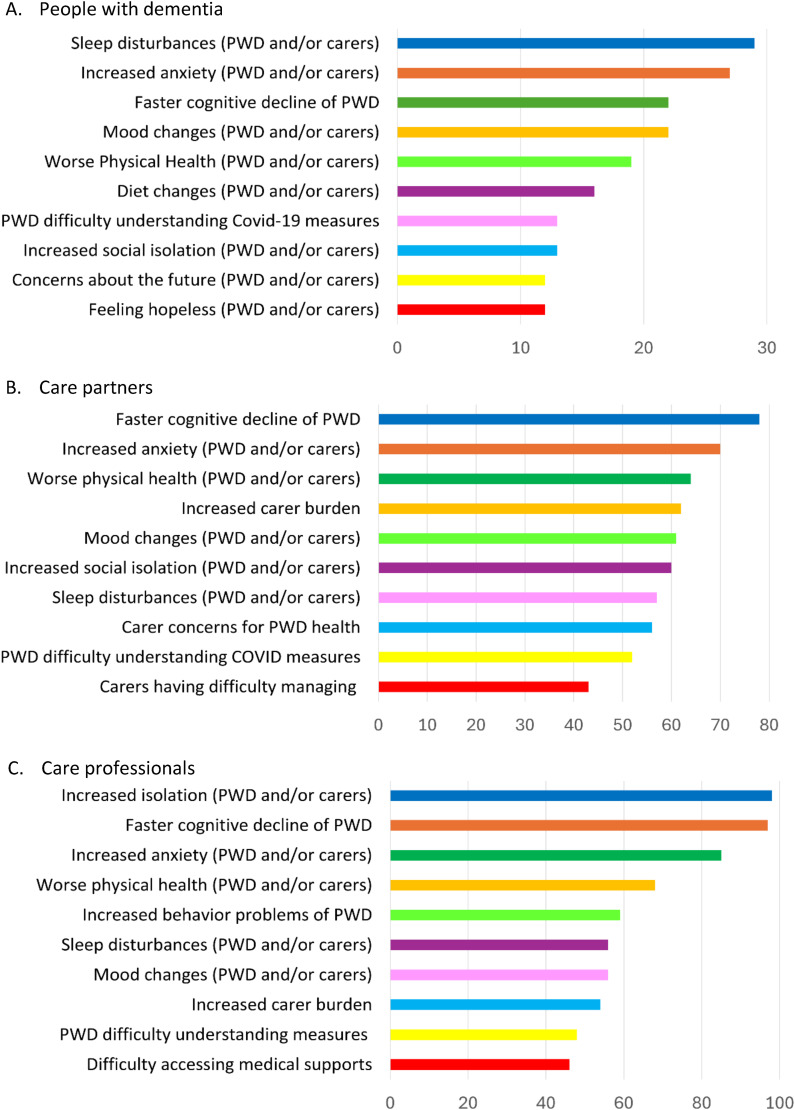
Top 10 priorities for (A) people with dementia (PWD), (B) caregivers (carers), and (C) care professionals.

Because there was substantial overlap between the priority lists of the countries, we describe the generic results here (Supplemental Table 3). However, we do want to point out that there were some country differences in priorities as specified in Supplemental Table 4. For example, ‘diet or appetite changes’ scored high in Ecuador, Nepal, Nigeria and Peru but not in other countries; and ‘financial uncertainties/difficulties’ scored high in India, Nigeria and South Africa, but not in the other countries. Across the countries, PWD were particularly concerned about the disruptive impact of the control measures on their daily routines (i.e., sleep, eating patterns, and social activities) and the subsequent impact on disease progression and overall physical and mental health (Figure 1A). Caregivers were mostly concerned about disease progression and the mental and physical health of the person with dementia (Figure 1B). They also struggled with dealing with the PWD not understanding the situation and the need for control measures. Moreover, having to deal with the control measures (e.g., maintaining hygiene and distance) and the consequences of the control measures (e.g., closure of day care centers) further added to the care responsibilities and perceived care burden. Care professionals felt that the control measures accelerated disease progression and worsened physical and mental health, possibly due to social isolation and disruption of social activities (Figure 1C). They also worried about the impact of control measures on the care burden for the caregivers.

The x-axis presents the number of votes. Each survey participant was asked to first prioritize 10 topics and then select a top 3. Each prioritized topic was given one vote, with an additional vote if the topic was also selected in the top 3.

### Step 3: Translating priorities into a research agenda

**Figure 2.**
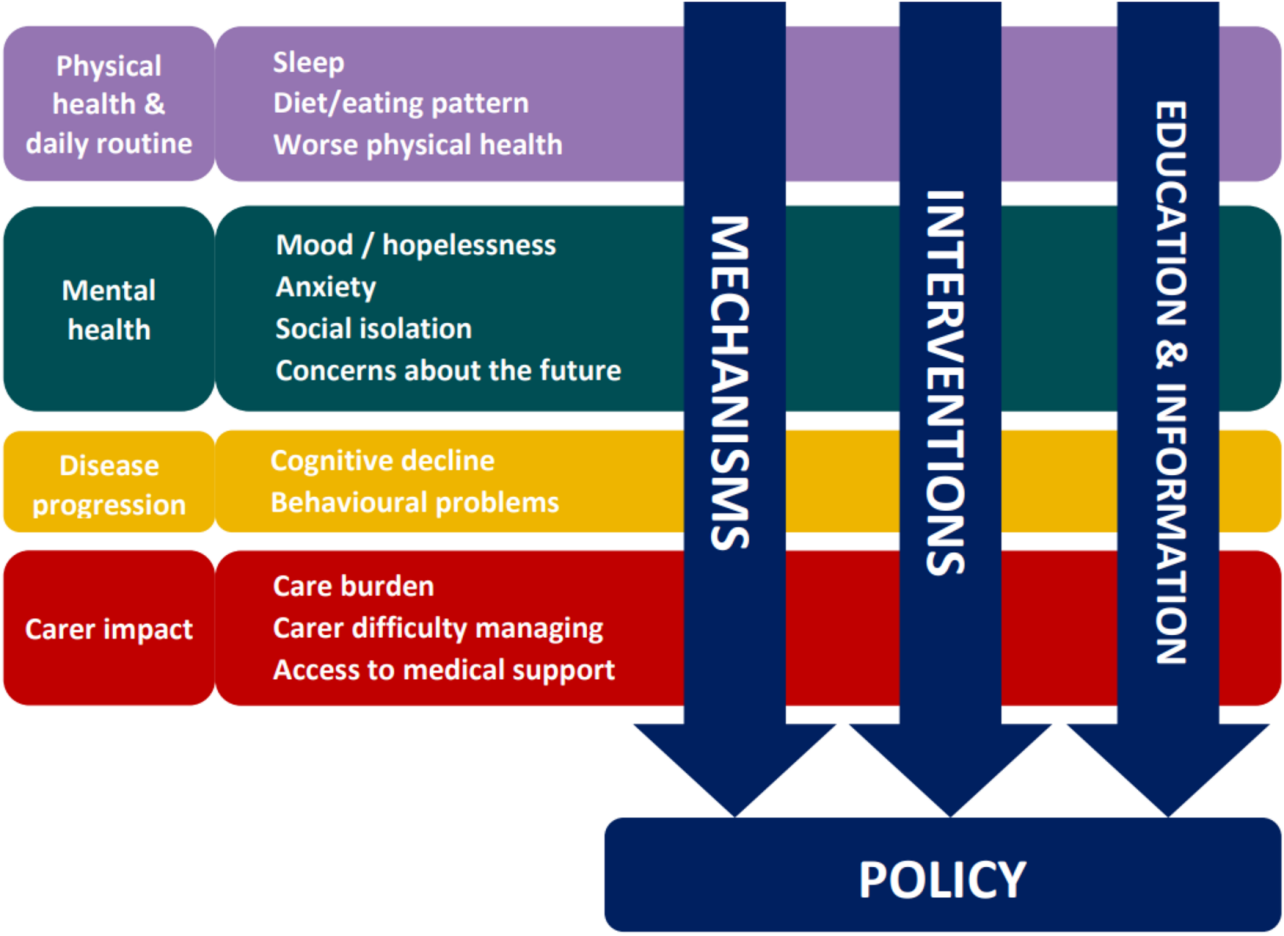
Matrix summarizing research agenda.

Given the large degree of overlap in prioritized topics across the three stakeholder groups (Supplemental Table 3), we decided to combine these topics into one generic list of 12 priorities. These priorities cluster into four themes: physical health and daily routine, mental health, disease progression, and carer impact (Figure 2). Looking at the prioritized topics, the consortium discussed which levels of knowledge were required to address the topics. A consensus fell on three pillars that are relevant for all prioritized topics:

1. *Understanding mechanisms*: This pillar involves research aimed at understanding the mechanisms (i.e., the causal pathways or mediating processes) by which the control measures affected PWD and their caregivers. Understanding these mechanisms is needed to subsequently develop effective interventions.
2. *Developing and evaluating interventions*: By interventions we mean any type of solution to reduce the impact of the control measures on PWD and their caregivers. These may include solutions on the policy level (e.g., alternate control measures or exemptions to control measures), solutions on the health services level (e.g., different ways of providing services or strategies to mitigate the impact of the control measures) and solutions on the informal care and support level (e.g., solutions to strengthen the resilience of the personal network of the person with dementia and care partner).
3. *Education and information*: Future research should focus both on what information or educational resources to provide, and how to provide it. This research is needed to develop equitable and culturally appropriate strategies that help knowledge users to understand, interpret, and apply advice in prevention guidance, healthcare access, and strategies to maintain resilience and daily routines.

These three pillars of research cut across the four themes and together form a matrix that summarizes the research agenda (Figure 2). Ultimately, outcomes of the proposed research should inform policy.

Chosen priorities were grouped into four themes. Three cross-cutting lines of research are recommended to (1) better understand the mechanisms underlying the negative impact of the control measures on the four theme's; (2) develop and evaluate interventions to mitigate these negative impacts of control measures; and (3) develop education and information materials to reduce the stress and worries related to the crisis. Ultimately outcomes of research should inform policy that more adequately addresses the needs of PWD and their caregivers.

## Discussion

This paper describes the process used to identify research priorities concerning the impact of COVID-19 control measures on PWD living at home and their caregivers. The resulting research agenda is both globally relevant and sensitive to local contexts, providing a solid foundation for informing policy and care in the planning for future public health emergencies. Successive waves of the coronavirus have left little doubt that it has transitioned from a pandemic to a seasonal infection like the seasonal flu. Nonetheless, we need to learn from the previous crisis to improve the support for PWD and their caregivers by meeting their actual needs in a future pandemic or crisis. Therefore, there is an urgency for funding agencies and researchers to act towards the proposed research agenda.

During the COVID-19 pandemic, policy makers prioritized suppressing spread of the virus over other needs.^12^ Looking back, this one-sided approach made it very difficult for people to meet their existing care needs. The findings from the survey reinforce this and indicate that people want measures that protect routine, mental health and social connection, and that minimize care burden (Figure 2). To inform policy that meets these needs, research is needed to better *understand the mechanisms* by which control measures affected people's lives. For example, the accelerated disease progression could be caused by social isolation and subsequently perceived loneliness and reduced social stimulation. Alternatively, the accelerated disease progression could also be caused by closure of day care facilities and subsequently not receiving physical and cognitive therapy. The two mechanisms may require different solutions to minimize the impact of the control measures on disease progression. Once the mechanism is understood, effective *interventions can be developed and evaluated* to mitigate the negative effects of the control measures. For example, during the COVID-19 pandemic, many care professionals switched from in-person care to telemedicine. It would be informative to evaluate how this support was received, how it could be improved, what other forms of communication would be useful, and how groups who do not have access to telemedicine can be reached.^13,14^ Both participants in published qualitative studies,^2,15–17^ and respondents to our survey expressed that not knowing how long the lockdown would go on for and uncertainty of what the future might bring caused extra stress and concerns on top of the existing care burden. Timely information and the prospect of an end date could have helped reduce the negative psychological effects of the lockdown. Therefore, research is needed to better understand the needs for *information* and best ways of communicating this information to avoid the stress caused by uncertainty.

The consistency in priorities across the countries suggests that the impact of the control measures had similar, mostly negative, effects on the health and wellbeing of PWD and their caregivers across Europe and the Global South, despite differences in health systems and cultures. However, there were some differences in prioritization that may be relevant to explore further and to take into account when actioning the research agenda in these specific countries. In India, Nigeria and South-Africa, ‘financial uncertainty’ and ‘lack of information on available services’ were prioritized, which should be viewed in the context of lower- and middle-income countries. In India, the COVID-19 pandemic had detrimental effects on livelihood and food security, provoking economic vulnerability.^18^ In Nigeria, health service costs are typically out-of-pocket, and access to quality healthcare services remains a significant challenge for many citizens. While the generic research agenda is also relevant for these countries, the economic context should be taken into account in future research and resulting recommendations for policy. In Brazil, ‘changes in quality of life’ ranked highly; in Chile, ‘experiencing grief’ ranked highly, and in Peru, ‘hopelessness’ ranked highly, suggesting a need for more emotional support with a focus on wellbeing in these countries. Further details per country can be found in Supplemental Table 4 of the policy report that describes the research agenda.^19^ An additional explanation of the (minor) differences in priorities across countries may be variations in the control measures. While control measures were largely consistent across countries, there were some variations, such as the exact distance that should be kept (varying between 1.5–2.5 meters) or the starting time of a curfew (if one was in place). The current data provide insufficient detail to tease this out further.

Concerns about accelerated disease progression was one of the top-rated priorities across all stakeholder groups (Figure 1). Studies that evaluated pre-lockdown functioning with peri- or post-lockdown functioning found mixed results, taking into account natural progression.^20–23^ Further research is needed to understand if the control measures indeed accelerated disease progression. If so, further research on mitigating factors is needed. If not, such information would be useful to reassure concerned PWD and caregivers.

The results of our priority setting exercise differ from the topics identified in earlier priority setting exercises, which covered priorities for research on dementia in general^24,25^ and were not specific for the COVID-19 pandemic. Most prior exercises were done within one country, but one included respondents from 39 countries^25^ and one systematic review summarized findings of 12 priority setting exercises.^24^ Unfortunately, these studies report little detail on country differences in prioritization, although the review concluded that low and middle income countries prioritize ‘awareness and education’, while high income countries prioritize ‘supporting people with dementia in their daily life’ and ‘addressing the needs of caregivers.^24^ Both studies noted that people from low and middle income countries are underrepresented in such priority setting exercises.^24,25^

Strengths of this research agenda include the cross-cultural scope, the co-design with stakeholder groups, and the mixed methods approach. The topics included in the priority setting survey were informed by a comprehensive literature review including data from 260,000 PWD and caregivers. In the survey, respondents had the opportunity to list additional topics. The few additional topics that were described, all matched with topics in the list. We are therefore confident that the most important topics and themes were identified.

A limitation of the study is that the numbers of respondents per country were relatively small. Particularly the number of PWD was small, partly due to ethical restrictions in disseminating the survey to these people in some countries. Therefore, findings may not generalize to the population level of each country. However, the priority setting process is considered a consultancy process of a more qualitative nature, in which representativeness and saturation are more important than sample size. Given the consistency of findings across countries and stakeholder groups, we believe that saturation was reached. Another limitation to consider is that in the priority setting process, we utilized elements from the James Lind Alliance protocol,^26^ but due to limited time, resources and the international nature of the study, it was not possible to follow all the protocolized steps. Ideally, the priority setting survey should have been followed by a focus group to finetune and decide on the final set of priorities. Given the many countries and languages involved, a single focus group in which stakeholders from each country are represented would not have been appropriate.

In conclusion, this paper describes the development of an evidence-based and stakeholder driven research agenda. By consulting PWD, caregivers and HCP from 14 countries across Europe and the Global South, we highlighted both shared and context-specific research priorities. PWD, their caregivers and HCP prioritize research to inform policy that protects routine, mental health and social connection, and that minimize care burden. The resulting agenda sets a solid foundation for guiding future research, health system responses, and policy development. Specific attention is needed for adequate education and information to reduce the stress caused by uncertainty about the future.

## Supplemental Material

sj-docx-1-alz-10.1177_13872877261451163 - Supplemental material for Setting research priorities around the impact of COVID-19 control measures on people with dementia and caregivers living at home: A 14-country perspective Supplemental material, sj-docx-1-alz-10.1177_13872877261451163 for Setting research priorities around the impact of COVID-19 control measures on people with dementia and caregivers living at home: A 14-country perspective by Geeske Peeters, Rachel L. Fitzpatrick, Tatyana Mollayeva, Yaohua Chen, Elisa de Paula França Resende, Tomas Leon, Lina M. Zapata-Restrepo, Kuripacha Tituaña, Magda Tsolaki, Faheem Arshad, Prekshya Thapa, Temitope Farombia, Belen Custodio, Khanyo Ntokozo Ngcobo, Roger O'Sullivan, Iracema Leroi and in Journal of Alzheimer's Disease
