# Supplementary material for: Setting research priorities around the impact of COVID-19 control measures on people with dementia and caregivers living at home: A 14-country perspective
Source: J Alzheimers Dis. 2026 May 30;112(1):471–82. doi: 10.1177/13872877261451163 (PMC13291391; doi:10.1177/13872877261451163)
Supplement: sj-docx-1-alz-10.1177_13872877261451163 - Supplemental material for Setting research priorities around the impact of COVID-19 control measures on people with dementia and caregivers living at home: A 14-country perspective [file sj-docx-1-alz-10.1177_13872877261451163.docx]

**Supplemental Material**

**Setting research priorities around the impact of COVID-19 control measures on people with dementia and caregivers living at home: A 14-country perspective**

**Supplemental Table 1.** Country-specific information on ethical approval.

| Country | Ethics approval provided by |
| --- | --- |
| Brazil | Universidade Federal de Minas Gerais, CAAE number: 32259520.4.1001.5149 |
| Chile | Comité de Ética Científico from "Servicio de Salud Metropolitano Oriente (Approved 8 August 2023) |
| Colombia | Not required |
| Ecuador | Not required |
| France | Not required if only health care professionals were recruited* |
| Greece | Ethical Committee of the Greek Association of Alzheimer’s Disease and Related Disorders (Approved 9 January 2020; 20179/2025 AI) |
| India | National Institute Of Mental Health & Neuro Sciences (NIMHANS/42nd IEC (BS & NS DIV)/2023, 21 July 2023) |
| Ireland | Research ethics board of Trinity College Dublin (REB #2023) |
| Nepal | Not required |
| Netherlands | Not required* |
| Nigeria | UI/UCH ethics committee (approval number: UI/EC/23/0530, 23 October 2023) |
| Peru | Not required |
| South Africa | University of KwaZulu- Natal Biomedical research ethical committee (reference number: BREC/00006373/2023) |
| UK | Not required* |

*The priority setting process is considered a consultancy process, not a research project, with very low risk of harm for the person consulted. Therefore, no ethics approval is required.

**Supplemental Table 2.** Topics derived from the literature review

The below table presents the list of 72 topics derived from the literature review. From this list, a condensed list of 38 topics was selected for inclusion in the priority setting survey.

| **N** | **Original list of 72 topics** | **Reduced list of 38 topics** |
| --- | --- | --- |
| **1** | Diet/appetite changes | Diet or appetite changes of PWD and/or informal carers |
| **2** | Sleep disturbances of care partner | Sleep disturbances for PWD and/or informal carers |
| **3** | Sleep disturbances of PWD |  |
| **4** | Increased use/dose of medications | Increased use/dose of medications by PWD |
| **5** | Weight change | Worsening physical health of PWD and/or care partner |
| **6** | Poorer physical health |  |
| **7** | Increased incontinence |  |
| **8** | Worsening mobility/motor functions |  |
| **9** | Fatigue |  |
| **10** | Faster cognitive decline | Faster cognitive decline of PWD |
| **11** | Difficulties understanding situation | PWD has difficulties understanding the COVID-19 situation |
| **12** | Difficulty managing control measures in context of PWD not understanding | Care partners have difficulty managing COVID-19 control measures in context of PWD not understanding the situation |
| **13** | Worsening psychological symptoms |  |
| **14** | Increased anxiety/stress | Increased anxiety/stress of PWD and/or informal carers |
| **15** | Fear of COVID infection |  |
| **16** | fears of infecting PWD |  |
| **17** | Feeling of never endless pandemic | PWD and/or care partner feeling hopeless (e.g., feeling discouraged, loss of purpose, feeling the pandemic will never end) |
| **18** | Feeling hopeless |  |
| **19** | Grief | Experiencing grief (PWD and/or care partner) |
| 20 | Mood changes | Mood changes of PWD and/or care partner (e.g., increased depression, sadness, mood swings) |
| **21** | concerns about health of PWD | Concerns about health of PWD |
| **22** | worries of worsening dementia of PWD |  |
| **23** | concerns about health of care partner | Concerns about health of care partner |
| **24** | concerns about health of family/friends | PWD and/or care partner concerns about health of family and friends |
| **25** | Care partner fear of struggling to cope | Care partner fears of struggling to cope due to COVID-19 measures |
| **26** | Fear of reduced support |  |
| **27** | Fears/concerns about the future | PWD and/or care partner concerns about the future |
| **28** | Concerns about leaving the house |  |
| 29 | More behavioral problems | Increased behavioral problems of PWD |
| **30** | Higher/increased loneliness |  |
| **31** | Isolation | Increased social isolation of PWD and/or care partner |
| **32** | Decreased quality of life | Changes in quality of life and/or life satisfaction (positive/negative) of PWD and/or care partner |
| **33** | Decreased life satisfaction |  |
| **34** | Increased care burden/responsibilities | Increased care burden and/or care responsibilities of care partner due to COVID-19 measures |
| **35** | Struggling to cope | Positive/negative coping strategies used by PWD and/or care partner |
| **36** | Dysfunctional coping strategies |  |
| **37** | Beneficial coping strategies |  |
| 38 | Change in quality of relationship between PWD and care partner | Changes (positive/negative) in the quality of relationships for PWD and/or care partner |
| 39 | Increased family Issues |  |
| **40** | Not able to use technologies |  |
| **41** | not satisfied with technology |  |
| **42** | Regular telephone calls to friends | Changes (positive/negative) in social contacts of PDW and/or care partner |
| **43** | Need of connection with other care partners |  |
| **44** | Changes in social contacts |  |
| **45** | Increased stigma | Increased stigma experienced by care partners during COVID-19 pandemic |
| 46 | Loss/change of/in work | Loss of work/change in work of care partner due to COVID-19 measures |
| **47** | Loss/interruption of physical activities | Interruption of physical and/or social activities of PWD and/or care partners |
| 48 | Loss of routine |  |
| 49 | New rules and restrictions |  |
| **50** | Financial uncertainties/difficulties | Financial uncertainties/difficulties for PWD and/or care partners |
| **51** | concerns about financial security due to COVID |  |
| **52** | Increased care time | Increased time care partner spend caring for PWD |
| **53** | Lack of respite time |  |
| **54** | Reduced personal care | PWD having reduced personal care |
| **55** | Needs vary per person, depending on personal situation |  |
| **56** | Emergence of new care needs |  |
| **57** | Guilt among family around being unable to visit PWD | Care partner experiencing guilt over not being able to visit PWD |
| **58** | Difficulties in accessing basic needs | Difficulty in accessing basic needs (e.g., difficulty going shopping, difficulties accessing care, etc.) |
| 59 | Loss of formal home care service |  |
| **60** | Discontinuation/shut down of services | Discontinuation/shut down of services (e.g., appointments, doctor visits, etc.) |
| **61** | Lack of access to medical support | Lack of access to medical support |
| **62** | Alternative care/support methods |  |
| **63** | Increased cost of care | Increased cost of care |
| **64** | Increased inequalities that existed before COVID | Increased inequities that existed before COVID-19 |
| **65** | Avoiding seeking help for health issues | PWD and/or care partner avoiding seeking help for health issues out of fear of getting COVID-19 in hospitals or doctor surgeries |
| **66** | Introversion/avoidance |  |
| **67** | Children of PWD taking over carer role |  |
| **68** | Reduced friend/family support | Reduced support for PWD and/or care partner |
| **69** | Need/want more support |  |
| **70** | Unable to stay during hospitalization for carers |  |
| **71** | Loss of independency |  |
| **72** | Lack of information | Lack of information on what supports and services were available for PWD and/or their care partners during the COVID-19 pandemic |

**Supplemental Table 3.** Generic list of prioritized topics in each of the stakeholder groups

|  |  | Generic list | | |
| --- | --- | --- | --- | --- |
|  | Overall | PWD | Carer | Professional |
| Diet or appetite changes of PWD and/or CP | 89 | 16 | 38 | 35 |
| Sleep disturbances for PWD and/or CP | 142 | 29 | 57 | 56 |
| Worsening physical health of PWD and/or CP | 151 | 19 | 64 | 68 |
| Faster cognitive decline of PWD | 197 | 22 | 78 | 97 |
| Increased use/dose of medications by PWD | 47 | 8 | 11 | 28 |
| PWD has difficulty understanding the COVID-19 situation | 113 | 13 | 52 | 48 |
| CP have difficulty managing COVID-19 control measures in context of PWD not understanding the situation | 85 | 3 | 43 | 39 |
| Increased anxiety/stress of PWD and/or care partners | 182 | 27 | 70 | 85 |
| PWD and/or CP feeling hopeless (e.g., feeling discouraged, loss of purpose, feeling the pandemic will never end) | 74 | 12 | 23 | 39 |
| Mood changes of PWD and/or CP (e.g., increased depression, sadness, mood swings) | 139 | 22 | 61 | 56 |
| Experiencing grief (PWD and/or CP) | 55 | 6 | 10 | 39 |
| Concerns about health of PWD | 98 | 9 | 56 | 33 |
| Concerns about health of CP | 51 | 1 | 33 | 17 |
| PWD and/or CP concerns about health of family and friends | 12 | 4 | 3 | 5 |
| CP fears of struggling to cope due to COVID-19 measures | 34 | 1 | 17 | 16 |
| PWD and/or CP concerns about the future | 46 | 12 | 17 | 17 |
| Increased behavioral problems of PWD | 111 | 11 | 41 | 59 |
| Increased social isolation of PWD and/or CP | 171 | 13 | 60 | 98 |
| Changes in quality of life and/or life satisfaction of PWD and/or CP | 83 | 8 | 41 | 34 |
| Increased care burden and/or responsibilities of CP | 119 | 3 | 62 | 54 |
| Positive/negative coping strategies used by PWD and/or CP | 32 | 2 | 14 | 16 |
| Changes in the quality of relationships for PWD and/or CP | 47 | 6 | 22 | 19 |
| Changes in social contacts of PWD and/or CP | 58 | 3 | 34 | 21 |
| Increased stigma experienced by CP during COVID-19 pandemic | 7 | 0 | 1 | 6 |
| Loss of work/change in work of CP due to COVID-19 measures | 34 | 1 | 20 | 13 |
| Interruption of physical and/or social activities of PWD and/or CP | 57 | 3 | 25 | 29 |
| PWD having reduced personal care | 39 | 4 | 17 | 18 |
| Increased time CP spend caring for person with dementia | 46 | 1 | 28 | 17 |
| Financial uncertainties/difficulties for PWD and/or CP | 53 | 9 | 32 | 12 |
| CP experiencing guilt over not being able to visit PWD | 22 | 2 | 8 | 12 |
| Difficulty in accessing basic needs (e.g., shopping, accessing care) | 51 | 6 | 19 | 26 |
| Discontinuation/shut down of services (e.g., doctor visits) | 74 | 7 | 27 | 40 |
| Lack of access to medical support | 68 | 6 | 16 | 46 |
| Increased cost of care | 34 | 2 | 16 | 16 |
| Reduced support for PWD and/or CP | 62 | 3 | 25 | 34 |
| Increased inequities that existed before COVID-19 | 20 | 0 | 4 | 16 |
| PWD and/or CP avoiding seeking help for health issues out of fear of getting COVID-19 in hospitals or doctor surgeries | 65 | 11 | 17 | 37 |
| Lack of information on what supports and services were available for PWD and/or CP during the COVID-19 pandemic | 64 | 3 | 38 | 23 |

Presented are the number of votes per topic; the top 10 per group is highlighted in yellow.

**Supplemental Table 4.** Country-specific results

Tables with country-specific results are provided below for each of the participating countries in alphabetical order. For each country, results are compared with the generic list of priorities. Highlighted in yellow are the topics with the most votes within each stakeholder group

**Brazil**

|  | Generic List | Brazil | | | |
| --- | --- | --- | --- | --- | --- |
|  |  | Overall | PWD | Carer | Professional |
| Diet or appetite changes of PWD and/or CP | 89 | 0 | 0 | 0 | 0 |
| Sleep disturbances for PWD and/or CP | 142 | 5 | 0 | 5 | 0 |
| Worsening physical health of PWD and/or CP | 151 | 11 | 0 | 7 | 4 |
| Faster cognitive decline of PWD | 197 | 16 | 2 | 9 | 5 |
| Increased use/dose of medications by PWD | 47 | 1 | 0 | 1 | 0 |
| PWD has difficulty understanding the COVID-19 situation | 113 | 7 | 0 | 7 | 0 |
| CP have difficulty managing COVID-19 control measures in context of PWD not understanding the situation | 85 | 6 | 0 | 4 | 2 |
| Increased anxiety/stress of PWD and/or care partners | 182 | 10 | 0 | 7 | 3 |
| PWD and/or CP feeling hopeless (e.g., feeling discouraged, loss of purpose, feeling the pandemic will never end) | 74 | 4 | 0 | 3 | 1 |
| Mood changes of PWD and/or CP (e.g., increased depression, sadness, mood swings) | 139 | 7 | 2 | 4 | 1 |
| Experiencing grief (PWD and/or CP) | 55 | 1 | 0 | 1 | 0 |
| Concerns about health of PWD | 98 | 11 | 0 | 10 | 1 |
| Concerns about health of CP | 51 | 7 | 0 | 7 | 0 |
| PWD and/or CP concerns about health of family and friends | 12 | 3 | 0 | 3 | 0 |
| CP fears of struggling to cope due to COVID-19 measures | 34 | 4 | 0 | 4 | 0 |
| PWD and/or CP concerns about the future | 46 | 2 | 1 | 1 | 0 |
| Increased behavioral problems of PWD | 111 | 11 | 0 | 5 | 6 |
| Increased social isolation of PWD and/or CP | 171 | 11 | 2 | 6 | 3 |
| Changes in quality of life and/or life satisfaction of PWD and/or CP | 83 | 5 | 2 | 3 | 0 |
| Increased care burden and/or responsibilities of CP | 119 | 15 | 1 | 10 | 4 |
| Positive/negative coping strategies used by PWD and/or CP | 32 | 1 | 0 | 1 | 0 |
| Changes in the quality of relationships for PWD and/or CP | 47 | 5 | 2 | 3 | 0 |
| Changes in social contacts of PWD and/or CP | 58 | 4 | 0 | 4 | 0 |
| Increased stigma experienced by CP during COVID-19 pandemic | 7 | 1 | 0 | 0 | 1 |
| Loss of work/change in work of CP due to COVID-19 measures | 34 | 3 | 0 | 0 | 3 |
| Interruption of physical and/or social activities of PWD and/or CP | 57 | 10 | 2 | 6 | 2 |
| PWD having reduced personal care | 39 | 2 | 0 | 2 | 0 |
| Increased time CP spend caring for person with dementia | 46 | 5 | 0 | 4 | 1 |
| Financial uncertainties/difficulties for PWD and/or CP | 53 | 4 | 0 | 1 | 3 |
| CP experiencing guilt over not being able to visit PWD | 22 | 1 | 1 | 0 | 0 |
| Difficulty accessing basic needs (e.g., shopping, accessing care) | 51 | 5 | 0 | 3 | 2 |
| Discontinuation/shut down of services (e.g., doctor visits) | 74 | 5 | 1 | 1 | 3 |
| Lack of access to medical support | 68 | 1 | 0 | 1 | 0 |
| Increased cost of care | 34 | 0 | 0 | 0 | 0 |
| Reduced support for PWD and/or CP | 62 | 5 | 0 | 3 | 2 |
| Increased inequities that existed before COVID-19 | 20 | 4 | 0 | 1 | 3 |
| PWD and/or CP avoiding seeking help for health issues out of fear of getting COVID-19 in hospitals or doctor surgeries | 65 | 5 | 1 | 3 | 1 |
| Lack of information on what supports and services were available for PWD and/or CP during the COVID-19 pandemic | 64 | 6 | 0 | 6 | 0 |

CP: care partner; PWD: person with dementia

**Chile**

|  | Generic List | Chile | | | |
| --- | --- | --- | --- | --- | --- |
|  |  | Overall | PWD | Carer | Professional |
| Diet or appetite changes of PWD and/or CP | 89 | 4 | 0 | 4 | 0 |
| Sleep disturbances for PWD and/or CP | 142 | 10 | 2 | 7 | 1 |
| Worsening physical health of PWD and/or CP | 151 | 14 | 0 | 14 | 0 |
| Faster cognitive decline of PWD | 197 | 16 | 2 | 13 | 1 |
| Increased use/dose of medications by PWD | 47 | 3 | 0 | 3 | 0 |
| PWD has difficulty understanding the COVID-19 situation | 113 | 12 | 0 | 11 | 1 |
| CP have difficulty managing COVID-19 control measures in context of PWD not understanding the situation | 85 | 5 | 0 | 5 | 0 |
| Increased anxiety/stress of PWD and/or care partners | 182 | 11 | 1 | 10 | 0 |
| PWD and/or CP feeling hopeless (e.g., feeling discouraged, loss of purpose, feeling the pandemic will never end) | 74 | 3 | 1 | 2 | 0 |
| Mood changes of PWD and/or CP (e.g., increased depression, sadness, mood swings) | 139 | 13 | 2 | 11 | 0 |
| Experiencing grief (PWD and/or CP) | 55 | 1 | 0 | 1 | 0 |
| Concerns about health of PWD | 98 | 12 | 1 | 10 | 1 |
| Concerns about health of CP | 51 | 8 | 0 | 8 | 0 |
| PWD and/or CP concerns about health of family and friends | 12 | 3 | 1 | 2 | 0 |
| CP fears of struggling to cope due to COVID-19 measures | 34 | 2 | 0 | 1 | 1 |
| PWD and/or CP concerns about the future | 46 | 8 | 1 | 7 | 0 |
| Increased behavioral problems of PWD | 111 | 7 | 1 | 6 | 0 |
| Increased social isolation of PWD and/or CP | 171 | 8 | 0 | 6 | 2 |
| Changes in quality of life and/or life satisfaction of PWD and/or CP | 83 | 7 | 0 | 7 | 0 |
| Increased care burden and/or responsibilities of CP | 119 | 10 | 0 | 8 | 2 |
| Positive/negative coping strategies used by PWD and/or CP | 32 | 5 | 0 | 5 | 0 |
| Changes in the quality of relationships for PWD and/or CP | 47 | 3 | 0 | 3 | 0 |
| Changes in social contacts of PWD and/or CP | 58 | 4 | 0 | 4 | 0 |
| Increased stigma experienced by CP during COVID-19 pandemic | 7 | 0 | 0 | 0 | 0 |
| Loss of work/change in work of CP due to COVID-19 measures | 34 | 5 | 0 | 5 | 0 |
| Interruption of physical and/or social activities of PWD and/or CP | 57 | 6 | 0 | 6 | 0 |
| PWD having reduced personal care | 39 | 1 | 1 | 0 | 0 |
| Increased time CP spend caring for person with dementia | 46 | 4 | 0 | 4 | 0 |
| Financial uncertainties/difficulties for PWD and/or CP | 53 | 4 | 0 | 4 | 0 |
| CP experiencing guilt over not being able to visit PWD | 22 | 0 | 0 | 0 | 0 |
| Difficulty accessing basic needs (e.g., shopping, accessing care) | 51 | 4 | 0 | 3 | 1 |
| Discontinuation/shut down of services (e.g., doctor visits) | 74 | 3 | 0 | 1 | 2 |
| Lack of access to medical support | 68 | 0 | 0 | 0 | 0 |
| Increased cost of care | 34 | 3 | 0 | 3 | 0 |
| Reduced support for PWD and/or CP | 62 | 4 | 0 | 4 | 0 |
| Increased inequities that existed before COVID-19 | 20 | 0 | 0 | 0 | 0 |
| PWD and/or CP avoiding seeking help for health issues out of fear of getting COVID-19 in hospitals or doctor surgeries | 65 | 2 | 0 | 1 | 1 |
| Lack of information on what supports and services were available for PWD and/or CP during the COVID-19 pandemic | 64 | 3 | 0 | 3 | 0 |

CP: care partner; PWD: person with dementia

**Colombia**

|  | Generic List | Colombia |
| --- | --- | --- |
|  |  | Professional |
| Diet or appetite changes of PWD and/or CP | 89 | 1 |
| Sleep disturbances for PWD and/or CP | 142 | 6 |
| Worsening physical health of PWD and/or CP | 151 | 3 |
| Faster cognitive decline of PWD | 197 | 9 |
| Increased use/dose of medications by PWD | 47 | 2 |
| PWD has difficulty understanding the COVID-19 situation | 113 | 3 |
| CP have difficulty managing COVID-19 control measures in context of PWD not understanding the situation | 85 | 1 |
| Increased anxiety/stress of PWD and/or care partners | 182 | 4 |
| PWD and/or CP feeling hopeless (e.g., feeling discouraged, loss of purpose, feeling the pandemic will never end) | 74 | 0 |
| Mood changes of PWD and/or CP (e.g., increased depression, sadness, mood swings) | 139 | 3 |
| Experiencing grief (PWD and/or CP) | 55 | 2 |
| Concerns about health of PWD | 98 | 1 |
| Concerns about health of CP | 51 | 0 |
| PWD and/or CP concerns about health of family and friends | 12 | 0 |
| CP fears of struggling to cope due to COVID-19 measures | 34 | 2 |
| PWD and/or CP concerns about the future | 46 | 3 |
| Increased behavioral problems of PWD | 111 | 0 |
| Increased social isolation of PWD and/or CP | 171 | 5 |
| Changes in quality of life and/or life satisfaction of PWD and/or CP | 83 | 2 |
| Increased care burden and/or responsibilities of CP | 119 | 4 |
| Positive/negative coping strategies used by PWD and/or CP | 32 | 1 |
| Changes in the quality of relationships for PWD and/or CP | 47 | 0 |
| Changes in social contacts of PWD and/or CP | 58 | 1 |
| Increased stigma experienced by CP during COVID-19 pandemic | 7 | 1 |
| Loss of work/change in work of CP due to COVID-19 measures | 34 | 1 |
| Interruption of physical and/or social activities of PWD and/or CP | 57 | 2 |
| PWD having reduced personal care | 39 | 0 |
| Increased time CP spend caring for person with dementia | 46 | 1 |
| Financial uncertainties/difficulties for PWD and/or CP | 53 | 1 |
| CP experiencing guilt over not being able to visit PWD | 22 | 0 |
| Difficulty in accessing basic needs (e.g., shopping, accessing care) | 51 | 2 |
| Discontinuation/shut down of services (e.g., doctor visits) | 74 | 1 |
| Lack of access to medical support | 68 | 3 |
| Increased cost of care | 34 | 2 |
| Reduced support for PWD and/or CP | 62 | 1 |
| Increased inequities that existed before COVID-19 | 20 | 1 |
| PWD and/or CP avoiding seeking help for health issues out of fear of getting COVID-19 in hospitals or doctor surgeries | 65 | 1 |
| Lack of information on what supports and services were available for PWD and/or CP during the COVID-19 pandemic | 64 | 1 |

CP: care partner; PWD: person with dementia

**Ecuador**

|  | Generic List | Ecuador | | |
| --- | --- | --- | --- | --- |
|  |  | Overall | Carer | Professional |
| Diet or appetite changes of PWD and/or CP | 89 | 15 | 3 | 12 |
| Sleep disturbances for PWD and/or CP | 142 | 23 | 4 | 18 |
| Worsening physical health of PWD and/or CP | 151 | 21 | 5 | 15 |
| Faster cognitive decline of PWD | 197 | 12 | 2 | 9 |
| Increased use/dose of medications by PWD | 47 | 9 | 0 | 6 |
| PWD has difficulty understanding the COVID-19 situation | 113 | 15 | 2 | 13 |
| CP have difficulty managing COVID-19 control measures in context of PWD not understanding the situation | 85 | 11 | 2 | 8 |
| Increased anxiety/stress of PWD and/or care partners | 182 | 27 | 4 | 22 |
| PWD and/or CP feeling hopeless (e.g., feeling discouraged, loss of purpose, feeling the pandemic will never end) | 74 | 13 | 1 | 11 |
| Mood changes of PWD and/or CP (e.g., increased depression, sadness, mood swings) | 139 | 18 | 2 | 13 |
| Experiencing grief (PWD and/or CP) | 55 | 13 | 1 | 12 |
| Concerns about health of PWD | 98 | 8 | 1 | 7 |
| Concerns about health of CP | 51 | 5 | 0 | 5 |
| PWD and/or CP concerns about health of family and friends | 12 | 6 | 2 | 4 |
| CP fears of struggling to cope due to COVID-19 measures | 34 | 3 | 1 | 2 |
| PWD and/or CP concerns about the future | 46 | 4 | 1 | 3 |
| Increased behavioral problems of PWD | 111 | 8 | 0 | 7 |
| Increased social isolation of PWD and/or CP | 171 | 16 | 4 | 12 |
| Changes in quality of life and/or life satisfaction of PWD and/or CP | 83 | 9 | 0 | 7 |
| Increased care burden and/or responsibilities of CP | 119 | 9 | 1 | 7 |
| Positive/negative coping strategies used by PWD and/or CP | 32 | 5 | 0 | 4 |
| Changes in the quality of relationships for PWD and/or CP | 47 | 3 | 0 | 3 |
| Changes in social contacts of PWD and/or CP | 58 | 2 | 0 | 2 |
| Increased stigma experienced by CP during COVID-19 pandemic | 7 | 1 | 0 | 1 |
| Loss of work/change in work of CP due to COVID-19 measures | 34 | 6 | 2 | 3 |
| Interruption of physical and/or social activities of PWD and/or CP | 57 | 3 | 1 | 2 |
| PWD having reduced personal care | 39 | 3 | 1 | 2 |
| Increased time CP spend caring for person with dementia | 46 | 2 | 1 | 1 |
| Financial uncertainties/difficulties for PWD and/or CP | 53 | 2 | 2 | 0 |
| CP experiencing guilt over not being able to visit PWD | 22 | 0 | 0 | 0 |
| Difficulty accessing basic needs (e.g., shopping, accessing care) | 51 | 6 | 1 | 5 |
| Discontinuation/shut down of services (e.g., doctor visits) | 74 | 2 | 1 | 1 |
| Lack of access to medical support | 68 | 10 | 0 | 8 |
| Increased cost of care | 34 | 11 | 4 | 7 |
| Reduced support for PWD and/or CP | 62 | 7 | 0 | 6 |
| Increased inequities that existed before COVID-19 | 20 | 4 | 0 | 4 |
| PWD and/or CP avoiding seeking help for health issues out of fear of getting COVID-19 in hospitals or doctor surgeries | 65 | 6 | 0 | 4 |
| Lack of information on what supports and services were available for PWD and/or CP during the COVID-19 pandemic | 64 | 8 | 2 | 5 |

CP: care partner; PWD: person with dementia

**France**

|  | Generic List | France | | |
| --- | --- | --- | --- | --- |
|  |  | Overall | Carer | Professional |
| Diet or appetite changes of PWD and/or CP | 89 | 1 | 1 | 0 |
| Sleep disturbances for PWD and/or CP | 142 | 2 | 1 | 1 |
| Worsening physical health of PWD and/or CP | 151 | 1 | 1 | 0 |
| Faster cognitive decline of PWD | 197 | 7 | 2 | 5 |
| Increased use/dose of medications by PWD | 47 | 2 | 0 | 2 |
| PWD has difficulty understanding the COVID-19 situation | 113 | 3 | 1 | 2 |
| CP have difficulty managing COVID-19 control measures in context of PWD not understanding the situation | 85 | 1 | 0 | 1 |
| Increased anxiety/stress of PWD and/or care partners | 182 | 7 | 2 | 5 |
| PWD and/or CP feeling hopeless (e.g., feeling discouraged, loss of purpose, feeling the pandemic will never end) | 74 | 1 | 0 | 1 |
| Mood changes of PWD and/or CP (e.g., increased depression, sadness, mood swings) | 139 | 2 | 2 | 0 |
| Experiencing grief (PWD and/or CP) | 55 | 4 | 0 | 4 |
| Concerns about health of PWD | 98 | 0 | 0 | 0 |
| Concerns about health of CP | 51 | 0 | 0 | 0 |
| PWD and/or CP concerns about health of family and friends | 12 | 0 | 0 | 0 |
| CP fears of struggling to cope due to COVID-19 measures | 34 | 1 | 0 | 1 |
| PWD and/or CP concerns about the future | 46 | 1 | 1 | 0 |
| Increased behavioral problems of PWD | 111 | 6 | 0 | 6 |
| Increased social isolation of PWD and/or CP | 171 | 12 | 6 | 6 |
| Changes in quality of life and/or life satisfaction of PWD and/or CP | 83 | 5 | 3 | 2 |
| Increased care burden and/or responsibilities of CP | 119 | 2 | 1 | 1 |
| Positive/negative coping strategies used by PWD and/or CP | 32 | 1 | 0 | 1 |
| Changes in the quality of relationships for PWD and/or CP | 47 | 1 | 1 | 0 |
| Changes in social contacts of PWD and/or CP | 58 | 2 | 2 | 0 |
| Increased stigma experienced by CP during COVID-19 pandemic | 7 | 0 | 0 | 0 |
| Loss of work/change in work of CP due to COVID-19 measures | 34 | 0 | 0 | 0 |
| Interruption of physical and/or social activities of PWD and/or CP | 57 | 5 | 3 | 2 |
| PWD having reduced personal care | 39 | 2 | 2 | 0 |
| Increased time CP spend caring for person with dementia | 46 | 7 | 3 | 4 |
| Financial uncertainties/difficulties for PWD and/or CP | 53 | 0 | 0 | 0 |
| CP experiencing guilt over not being able to visit PWD | 22 | 1 | 0 | 1 |
| Difficulty accessing basic needs (e.g., shopping, accessing care) | 51 | 1 | 0 | 1 |
| Discontinuation/shut down of services (e.g., doctor visits) | 74 | 4 | 0 | 4 |
| Lack of access to medical support | 68 | 2 | 0 | 2 |
| Increased cost of care | 34 | 0 | 0 | 0 |
| Reduced support for PWD and/or CP | 62 | 4 | 2 | 2 |
| Increased inequities that existed before COVID-19 | 20 | 1 | 0 | 1 |
| PWD and/or CP avoiding seeking help for health issues out of fear of getting COVID-19 in hospitals or doctor surgeries | 65 | 1 | 0 | 1 |
| Lack of information on what supports and services were available for PWD and/or CP during the COVID-19 pandemic | 64 | 1 | 1 | 0 |

CP: care partner; PWD: person with dementia

**Greece**

|  | Generic List | Greece | | |
| --- | --- | --- | --- | --- |
|  |  | Overall | Carer | Professional |
| Diet or appetite changes of PWD and/or CP | 89 | 1 | 1 | 0 |
| Sleep disturbances for PWD and/or CP | 142 | 6 | 4 | 2 |
| Worsening physical health of PWD and/or CP | 151 | 13 | 7 | 6 |
| Faster cognitive decline of PWD | 197 | 15 | 6 | 9 |
| Increased use/dose of medications by PWD | 47 | 3 | 1 | 2 |
| PWD has difficulty understanding the COVID-19 situation | 113 | 11 | 9 | 2 |
| CP have difficulty managing COVID-19 control measures in context of PWD not understanding the situation | 85 | 14 | 11 | 3 |
| Increased anxiety/stress of PWD and/or care partners | 182 | 21 | 10 | 11 |
| PWD and/or CP feeling hopeless (e.g., feeling discouraged, loss of purpose, feeling the pandemic will never end) | 74 | 3 | 1 | 2 |
| Mood changes of PWD and/or CP (e.g., increased depression, sadness, mood swings) | 139 | 16 | 7 | 9 |
| Experiencing grief (PWD and/or CP) | 55 | 4 | 2 | 2 |
| Concerns about health of PWD | 98 | 16 | 11 | 5 |
| Concerns about health of CP | 51 | 7 | 6 | 1 |
| PWD and/or CP concerns about health of family and friends | 12 | 3 | 2 | 1 |
| CP fears of struggling to cope due to COVID-19 measures | 34 | 2 | 1 | 1 |
| PWD and/or CP concerns about the future | 46 | 3 | 2 | 1 |
| Increased behavioral problems of PWD | 111 | 9 | 4 | 5 |
| Increased social isolation of PWD and/or CP | 171 | 19 | 8 | 11 |
| Changes in quality of life and/or life satisfaction of PWD and/or CP | 83 | 14 | 10 | 4 |
| Increased care burden and/or responsibilities of CP | 119 | 13 | 8 | 5 |
| Positive/negative coping strategies used by PWD and/or CP | 32 | 3 | 1 | 2 |
| Changes in the quality of relationships for PWD and/or CP | 47 | 1 | 1 | 0 |
| Changes in social contacts of PWD and/or CP | 58 | 6 | 5 | 1 |
| Increased stigma experienced by CP during COVID-19 pandemic | 7 | 0 | 0 | 0 |
| Loss of work/change in work of CP due to COVID-19 measures | 34 | 3 | 3 | 0 |
| Interruption of physical and/or social activities of PWD and/or CP | 57 | 19 | 10 | 9 |
| PWD having reduced personal care | 39 | 3 | 3 | 0 |
| Increased time CP spend caring for person with dementia | 46 | 5 | 2 | 3 |
| Financial uncertainties/difficulties for PWD and/or CP | 53 | 6 | 5 | 1 |
| CP experiencing guilt over not being able to visit PWD | 22 | 5 | 0 | 5 |
| Difficulty accessing basic needs (e.g., shopping, accessing care) | 51 | 4 | 2 | 2 |
| Discontinuation/shut down of services (e.g., doctor visits) | 74 | 8 | 5 | 3 |
| Lack of access to medical support | 68 | 12 | 7 | 5 |
| Increased cost of care | 34 | 5 | 5 | 0 |
| Reduced support for PWD and/or CP | 62 | 8 | 3 | 5 |
| Increased inequities that existed before COVID-19 | 20 | 1 | 1 | 0 |
| PWD and/or CP avoiding seeking help for health issues out of fear of getting COVID-19 in hospitals or doctor surgeries | 65 | 4 | 2 | 2 |
| Lack of information on what supports and services were available for PWD and/or CP during the COVID-19 pandemic | 64 | 4 | 2 | 2 |

CP: care partner; PWD: person with dementia

**India**

|  | Generic List | India | | |
| --- | --- | --- | --- | --- |
|  |  | Overall | Carer | Professional |
| Diet or appetite changes of PWD and/or CP | 89 | 2 | 2 | 0 |
| Sleep disturbances for PWD and/or CP | 142 | 2 | 1 | 1 |
| Worsening physical health of PWD and/or CP | 151 | 2 | 2 | 0 |
| Faster cognitive decline of PWD | 197 | 8 | 8 | 0 |
| Increased use/dose of medications by PWD | 47 | 1 | 0 | 1 |
| PWD has difficulty understanding the COVID-19 situation | 113 | 2 | 1 | 1 |
| CP have difficulty managing COVID-19 control measures in context of PWD not understanding the situation | 85 | 3 | 2 | 1 |
| Increased anxiety/stress of PWD and/or care partners | 182 | 4 | 4 | 0 |
| PWD and/or CP feeling hopeless (e.g., feeling discouraged, loss of purpose, feeling the pandemic will never end) | 74 | 2 | 1 | 1 |
| Mood changes of PWD and/or CP (e.g., increased depression, sadness, mood swings) | 139 | 4 | 4 | 0 |
| Experiencing grief (PWD and/or CP) | 55 | 0 | 0 | 0 |
| Concerns about health of PWD | 98 | 6 | 6 | 0 |
| Concerns about health of CP | 51 | 1 | 1 | 0 |
| PWD and/or CP concerns about health of family and friends | 12 | 0 | 0 | 0 |
| CP fears of struggling to cope due to COVID-19 measures | 34 | 4 | 4 | 0 |
| PWD and/or CP concerns about the future | 46 | 2 | 2 | 0 |
| Increased behavioral problems of PWD | 111 | 7 | 5 | 2 |
| Increased social isolation of PWD and/or CP | 171 | 6 | 4 | 2 |
| Changes in quality of life and/or life satisfaction of PWD and/or CP | 83 | 4 | 4 | 0 |
| Increased care burden and/or responsibilities of CP | 119 | 3 | 2 | 1 |
| Positive/negative coping strategies used by PWD and/or CP | 32 | 3 | 2 | 1 |
| Changes in the quality of relationships for PWD and/or CP | 47 | 1 | 1 | 0 |
| Changes in social contacts of PWD and/or CP | 58 | 5 | 4 | 1 |
| Increased stigma experienced by CP during COVID-19 pandemic | 7 | 0 | 0 | 0 |
| Loss of work/change in work of CP due to COVID-19 measures | 34 | 4 | 3 | 1 |
| Interruption of physical and/or social activities of PWD and/or CP | 57 | 0 | 0 | 0 |
| PWD having reduced personal care | 39 | 0 | 0 | 0 |
| Increased time CP spend caring for person with dementia | 46 |  | 3 | 1 |
| Financial uncertainties/difficulties for PWD and/or CP | 53 | 4 | 4 | 0 |
| CP experiencing guilt over not being able to visit PWD | 22 | 0 | 0 | 0 |
| Difficulty accessing basic needs (e.g., shopping, accessing care) | 51 | 1 | 0 | 1 |
| Discontinuation/shut down of services (e.g., doctor visits) | 74 | 2 | 1 | 1 |
| Lack of access to medical support | 68 | 0 | 0 | 0 |
| Increased cost of care | 34 | 0 | 0 | 0 |
| Reduced support for PWD and/or CP | 62 | 0 | 0 | 0 |
| Increased inequities that existed before COVID-19 | 20 | 0 | 0 | 0 |
| PWD and/or CP avoiding seeking help for health issues out of fear of getting COVID-19 in hospitals or doctor surgeries | 65 | 1 | 0 | 1 |
| Lack of information on what supports and services were available for PWD and/or CP during the COVID-19 pandemic | 64 | 4 | 3 | 1 |

CP: care partner; PWD: person with dementia

**Ireland**

|  | Generic List | Ireland | | | |
| --- | --- | --- | --- | --- | --- |
|  |  | Overall | PWD | Carer | Professional |
| Diet or appetite changes of PWD and/or CP | 89 | 5 | 0 | 3 | 2 |
| Sleep disturbances for PWD and/or CP | 142 | 12 | 3 | 7 | 2 |
| Worsening physical health of PWD and/or CP | 151 | 8 | 3 | 4 | 1 |
| Faster cognitive decline of PWD | 197 | 17 | 4 | 7 | 6 |
| Increased use/dose of medications by PWD | 47 | 5 | 2 | 1 | 2 |
| PWD has difficulty understanding the COVID-19 situation | 113 | 7 | 0 | 6 | 1 |
| CP have difficulty managing COVID-19 control measures in context of PWD not understanding the situation | 85 | 4 | 1 | 2 | 1 |
| Increased anxiety/stress of PWD and/or care partners | 182 | 8 | 3 | 4 | 1 |
| PWD and/or CP feeling hopeless (e.g., feeling discouraged, loss of purpose, feeling the pandemic will never end) | 74 | 8 | 2 | 3 | 3 |
| Mood changes of PWD and/or CP (e.g., increased depression, sadness, mood swings) | 139 | 13 | 1 | 8 | 4 |
| Experiencing grief (PWD and/or CP) | 55 | 4 | 2 | 0 | 2 |
| Concerns about health of PWD | 98 | 2 | 1 | 0 | 1 |
| Concerns about health of CP | 51 | 0 | 0 | 0 | 0 |
| PWD and/or CP concerns about health of family and friends | 12 | 3 | 0 | 2 | 1 |
| CP fears of struggling to cope due to COVID-19 measures | 34 | 4 | 1 | 3 | 0 |
| PWD and/or CP concerns about the future | 46 | 5 | 3 | 1 | 1 |
| Increased behavioral problems of PWD | 111 | 12 | 4 | 2 | 6 |
| Increased social isolation of PWD and/or CP | 171 | 16 | 3 | 6 | 7 |
| Changes in quality of life and/or life satisfaction of PWD and/or CP | 83 | 12 | 2 | 7 | 3 |
| Increased care burden and/or responsibilities of CP | 119 | 9 | 1 | 7 | 1 |
| Positive/negative coping strategies used by PWD and/or CP | 32 | 1 | 0 | 1 | 0 |
| Changes in the quality of relationships for PWD and/or CP | 47 | 9 | 4 | 3 | 2 |
| Changes in social contacts of PWD and/or CP | 58 | 2 | 0 | 1 | 1 |
| Increased stigma experienced by CP during COVID-19 pandemic | 7 | 0 | 0 | 0 | 0 |
| Loss of work/change in work of CP due to COVID-19 measures | 34 | 3 | 1 | 2 | 0 |
| Interruption of physical and/or social activities of PWD and/or CP | 57 | 14 | 2 | 5 | 7 |
| PWD having reduced personal care | 39 | 1 | 1 | 0 | 0 |
| Increased time CP spend caring for person with dementia | 46 | 0 | 0 | 0 | 0 |
| Financial uncertainties/difficulties for PWD and/or CP | 53 | 2 | 0 | 1 | 1 |
| CP experiencing guilt over not being able to visit PWD | 22 | 3 | 0 | 2 | 1 |
| Difficulty accessing basic needs (e.g., shopping, accessing care) | 51 | 6 | 1 | 2 | 3 |
| Discontinuation/shut down of services (e.g., doctor visits) | 74 | 8 | 0 | 2 | 6 |
| Lack of access to medical support | 68 | 9 | 2 | 1 | 6 |
| Increased cost of care | 34 | 1 | 0 | 0 | 1 |
| Reduced support for PWD and/or CP | 62 | 6 | 0 | 2 | 4 |
| Increased inequities that existed before COVID-19 | 20 | 0 | 0 | 0 | 0 |
| PWD and/or CP avoiding seeking help for health issues out of fear of getting COVID-19 in hospitals or doctor surgeries | 65 | 12 | 4 | 0 | 6 |
| Lack of information on what supports and services were available for PWD and/or CP during the COVID-19 pandemic | 64 | 10 | 1 | 8 | 1 |

CP: care partner; PWD: person with dementia

**Nepal**

|  | Generic List | Nepal | | |
| --- | --- | --- | --- | --- |
|  |  | Overall | Carer | Professional |
| Diet or appetite changes of PWD and/or CP | 89 | 10 | 1 | 9 |
| Sleep disturbances for PWD and/or CP | 142 | 9 | 6 | 3 |
| Worsening physical health of PWD and/or CP | 151 | 6 | 2 | 4 |
| Faster cognitive decline of PWD | 197 | 3 | 3 | 0 |
| Increased use/dose of medications by PWD | 47 | 1 | 1 | 0 |
| PWD has difficulty understanding the COVID-19 situation | 113 | 2 | 1 | 1 |
| CP have difficulty managing COVID-19 control measures in context of PWD not understanding the situation | 85 | 4 | 0 | 4 |
| Increased anxiety/stress of PWD and/or care partners | 182 | 12 | 4 | 8 |
| PWD and/or CP feeling hopeless (e.g., feeling discouraged, loss of purpose, feeling the pandemic will never end) | 74 | 4 | 0 | 4 |
| Mood changes of PWD and/or CP (e.g., increased depression, sadness, mood swings) | 139 | 6 | 1 | 5 |
| Experiencing grief (PWD and/or CP) | 55 | 4 | 0 | 4 |
| Concerns about health of PWD | 98 | 3 | 1 | 2 |
| Concerns about health of CP | 51 | 5 | 1 | 4 |
| PWD and/or CP concerns about health of family and friends | 12 | 0 | 0 | 0 |
| CP fears of struggling to cope due to COVID-19 measures | 34 | 1 | 1 | 0 |
| PWD and/or CP concerns about the future | 46 | 7 | 1 | 6 |
| Increased behavioral problems of PWD | 111 | 3 | 2 | 1 |
| Increased social isolation of PWD and/or CP | 171 | 2 | 0 | 2 |
| Changes in quality of life and/or life satisfaction of PWD and/or CP | 83 | 3 | 1 | 2 |
| Increased care burden and/or responsibilities of CP | 119 | 6 | 4 | 2 |
| Positive/negative coping strategies used by PWD and/or CP | 32 | 2 | 0 | 2 |
| Changes in the quality of relationships for PWD and/or CP | 47 | 5 | 2 | 3 |
| Changes in social contacts of PWD and/or CP | 58 | 5 | 2 | 3 |
| Increased stigma experienced by CP during COVID-19 pandemic | 7 | 2 | 0 | 2 |
| Loss of work/change in work of CP due to COVID-19 measures | 34 | 2 | 1 | 1 |
| Interruption of physical and/or social activities of PWD and/or CP | 57 | 0 | 0 | 0 |
| PWD having reduced personal care | 39 | 8 | 2 | 6 |
| Increased time CP spend caring for person with dementia | 46 | 0 | 0 | 0 |
| Financial uncertainties/difficulties for PWD and/or CP | 53 | 3 | 0 | 3 |
| CP experiencing guilt over not being able to visit PWD | 22 | 2 | 0 | 2 |
| Difficulty accessing basic needs (e.g., shopping, accessing care) | 51 | 2 | 0 | 2 |
| Discontinuation/shut down of services (e.g., doctor visits) | 74 | 5 | 3 | 2 |
| Lack of access to medical support | 68 | 8 | 2 | 6 |
| Increased cost of care | 34 | 3 | 1 | 2 |
| Reduced support for PWD and/or CP | 62 | 2 | 1 | 1 |
| Increased inequities that existed before COVID-19 | 20 | 0 | 0 | 0 |
| PWD and/or CP avoiding seeking help for health issues out of fear of getting COVID-19 in hospitals or doctor surgeries | 65 | 3 | 0 | 3 |
| Lack of information on what supports and services were available for PWD and/or CP during the COVID-19 pandemic | 64 | 4 | 1 | 3 |

CP: care partner; PWD: person with dementia

**Netherlands**

|  | Generic list | Netherlands | |
| --- | --- | --- | --- |
|  |  | Carer | Professional |
| Diet or appetite changes of PWD and/or CP | 89 | 4 | 0 |
| Sleep disturbances for PWD and/or CP | 142 | 1 | 0 |
| Worsening physical health of PWD and/or CP | 151 | 2 | 6 |
| Faster cognitive decline of PWD | 197 | 8 | 12 |
| Increased use/dose of medications by PWD | 47 | 0 | 1 |
| PWD has difficulty understanding the COVID-19 situation | 113 | 4 | 5 |
| CP have difficulty managing COVID-19 control measures in context of PWD not understanding the situation | 85 | 4 | 5 |
| Increased anxiety/stress of PWD and/or care partners | 182 | 1 | 0 |
| PWD and/or CP feeling hopeless (e.g., feeling discouraged, loss of purpose, feeling the pandemic will never end) | 74 | 2 | 1 |
| Mood changes of PWD and/or CP (e.g., increased depression, sadness, mood swings) | 139 | 5 | 3 |
| Experiencing grief (PWD and/or CP) | 55 | 0 | 3 |
| Concerns about health of PWD | 98 | 4 | 2 |
| Concerns about health of CP | 51 | 2 | 0 |
| PWD and/or CP concerns about health of family and friends | 12 | 2 | 1 |
| CP fears of struggling to cope due to COVID-19 measures | 34 | 1 | 1 |
| PWD and/or CP concerns about the future | 46 | 1 | 1 |
| Increased behavioral problems of PWD | 111 | 1 | 2 |
| Increased social isolation of PWD and/or CP | 171 | 9 | 11 |
| Changes in quality of life and/or life satisfaction of PWD and/or CP | 83 | 1 | 3 |
| Increased care burden and/or responsibilities of CP | 119 | 7 | 10 |
| Positive/negative coping strategies used by PWD and/or CP | 32 | 0 | 0 |
| Changes in the quality of relationships for PWD and/or CP | 47 | 1 | 3 |
| Changes in social contacts of PWD and/or CP | 58 | 5 | 5 |
| Increased stigma experienced by CP during COVID-19 pandemic | 7 | 0 | 0 |
| Loss of work/change in work of CP due to COVID-19 measures | 34 | 1 | 0 |
| Interruption of physical and/or social activities of PWD and/or CP | 57 | 2 | 6 |
| PWD having reduced personal care | 39 | 3 | 2 |
| Increased time CP spend caring for person with dementia | 46 | 1 | 0 |
| Financial uncertainties/difficulties for PWD and/or CP | 53 | 1 | 0 |
| CP experiencing guilt over not being able to visit PWD | 22 | 2 | 1 |
| Difficulty accessing basic needs (e.g., shopping, accessing care) | 51 | 0 | 3 |
| Discontinuation/shut down of services (e.g., doctor visits) | 74 | 3 | 1 |
| Lack of access to medical support | 68 | 0 | 2 |
| Increased cost of care | 34 | 0 | 0 |
| Reduced support for PWD and/or CP | 62 | 1 | 4 |
| Increased inequities that existed before COVID-19 | 20 | 0 | 1 |
| PWD and/or CP avoiding seeking help for health issues out of fear of getting COVID-19 in hospitals or doctor surgeries | 65 | 0 | 2 |
| Lack of information on what supports and services were available for PWD and/or CP during the COVID-19 pandemic | 64 | 3 | 1 |

CP: care partner; PWD: person with dementia

**Nigeria**

|  | Generic List | Nigeria | | | |
| --- | --- | --- | --- | --- | --- |
|  |  | Overall | PWD | Carer | Professional |
| Diet or appetite changes of PWD and/or CP | 89 | 10 | 2 | 2 | 6 |
| Sleep disturbances for PWD and/or CP | 142 | 14 | 5 | 3 | 6 |
| Worsening physical health of PWD and/or CP | 151 | 16 | 5 | 1 | 10 |
| Faster cognitive decline of PWD | 197 | 14 | 6 | 3 | 5 |
| Increased use/dose of medications by PWD | 47 | 10 | 2 | 0 | 8 |
| PWD has difficulty understanding the COVID-19 situation | 113 | 11 | 5 | 1 | 5 |
| CP have difficulty managing COVID-19 control measures in context of PWD not understanding the situation | 85 | 1 | 0 | 0 | 1 |
| Increased anxiety/stress of PWD and/or care partners | 182 | 21 | 10 | 2 | 9 |
| PWD and/or CP feeling hopeless (e.g., feeling discouraged, loss of purpose, feeling the pandemic will never end) | 74 | 7 | 2 | 2 | 3 |
| Mood changes of PWD and/or CP (e.g., increased depression, sadness, mood swings) | 139 | 11 | 4 | 3 | 4 |
| Experiencing grief (PWD and/or CP) | 55 | 7 | 2 | 2 | 3 |
| Concerns about health of PWD | 98 | 8 | 1 | 2 | 5 |
| Concerns about health of CP | 51 | 7 | 0 | 4 | 3 |
| PWD and/or CP concerns about health of family and friends | 12 | 0 | 0 | 0 | 0 |
| CP fears of struggling to cope due to COVID-19 measures | 34 | 4 | 0 | 0 | 4 |
| PWD and/or CP concerns about the future | 46 | 2 | 0 | 0 | 2 |
| Increased behavioral problems of PWD | 111 | 6 | 1 | 4 | 1 |
| Increased social isolation of PWD and/or CP | 171 | 9 | 1 | 3 | 5 |
| Changes in quality of life and/or life satisfaction of PWD and/or CP | 83 | 3 | 1 | 1 | 1 |
| Increased care burden and/or responsibilities of CP | 119 | 7 | 0 | 4 | 3 |
| Positive/negative coping strategies used by PWD and/or CP | 32 | 3 | 2 | 0 | 1 |
| Changes in the quality of relationships for PWD and/or CP | 47 | 6 | 0 | 2 | 4 |
| Changes in social contacts of PWD and/or CP | 58 | 4 | 1 | 3 | 0 |
| Increased stigma experienced by CP during COVID-19 pandemic | 7 | 1 | 0 | 1 | 0 |
| Loss of work/change in work of CP due to COVID-19 measures | 34 | 2 | 0 | 1 | 1 |
| Interruption of physical and/or social activities of PWD and/or CP | 57 | 0 | 0 | 0 | 0 |
| PWD having reduced personal care | 39 | 4 | 2 | 0 | 2 |
| Increased time CP spend caring for person with dementia | 46 | 5 | 0 | 5 | 0 |
| Financial uncertainties/difficulties for PWD and/or CP | 53 | 16 | 8 | 7 | 1 |
| CP experiencing guilt over not being able to visit PWD | 22 | 0 | 0 | 0 | 0 |
| Difficulty accessing basic needs (e.g., shopping, accessing care) | 51 | 6 | 3 | 1 | 2 |
| Discontinuation/shut down of services (e.g., doctor visits) | 74 | 11 | 2 | 3 | 6 |
| Lack of access to medical support | 68 | 9 | 2 | 3 | 4 |
| Increased cost of care | 34 | 3 | 2 | 0 | 1 |
| Reduced support for PWD and/or CP | 62 | 6 | 2 | 0 | 4 |
| Increased inequities that existed before COVID-19 | 20 | 1 | 0 | 0 | 1 |
| PWD and/or CP avoiding seeking help for health issues out of fear of getting COVID-19 in hospitals or doctor surgeries | 65 | 10 | 2 | 4 | 4 |
| Lack of information on what supports and services were available for PWD and/or CP during the COVID-19 pandemic | 64 | 4 | 1 | 2 | 1 |

CP: care partner; PWD: person with dementia

**Peru**

|  | Generic List | Peru | | | |
| --- | --- | --- | --- | --- | --- |
|  |  | Overall | PWD | Carer | Professional |
| Diet or appetite changes of PWD and/or CP | 89 | 22 | 11 | 11 | 0 |
| Sleep disturbances for PWD and/or CP | 142 | 26 | 13 | 10 | 3 |
| Worsening physical health of PWD and/or CP | 151 | 21 | 9 | 7 | 5 |
| Faster cognitive decline of PWD | 197 | 23 | 6 | 9 | 8 |
| Increased use/dose of medications by PWD | 47 | 8 | 4 | 2 | 2 |
| PWD has difficulty understanding the COVID-19 situation | 113 | 17 | 6 | 4 | 7 |
| CP have difficulty managing COVID-19 control measures in context of PWD not understanding the situation | 85 | 7 | 1 | 4 | 2 |
| Increased anxiety/stress of PWD and/or care partners | 182 | 24 | 8 | 9 | 7 |
| PWD and/or CP feeling hopeless (e.g., feeling discouraged, loss of purpose, feeling the pandemic will never end) | 74 | 11 | 3 | 5 | 3 |
| Mood changes of PWD and/or CP (e.g., increased depression, sadness, mood swings) | 139 | 15 | 6 | 5 | 4 |
| Experiencing grief (PWD and/or CP) | 55 | 4 | 0 | 3 | 1 |
| Concerns about health of PWD | 98 | 9 | 3 | 5 | 1 |
| Concerns about health of CP | 51 | 6 | 1 | 3 | 2 |
| PWD and/or CP concerns about health of family and friends | 12 | 5 | 4 | 1 | 0 |
| CP fears of struggling to cope due to COVID-19 measures | 34 | 1 | 0 | 0 | 1 |
| PWD and/or CP concerns about the future | 46 | 6 | 6 | 0 | 0 |
| Increased behavioral problems of PWD | 111 | 9 | 2 | 2 | 5 |
| Increased social isolation of PWD and/or CP | 171 | 6 | 2 | 0 | 4 |
| Changes in quality of life and/or life satisfaction of PWD and/or CP | 83 | 3 | 1 | 1 | 1 |
| Increased care burden and/or responsibilities of CP | 119 | 6 | 0 | 1 | 5 |
| Positive/negative coping strategies used by PWD and/or CP | 32 | 1 | 0 | 1 | 0 |
| Changes in the quality of relationships for PWD and/or CP | 47 | 0 | 0 | 0 | 0 |
| Changes in social contacts of PWD and/or CP | 58 | 1 | 0 | 0 | 1 |
| Increased stigma experienced by CP during COVID-19 pandemic | 7 | 0 | 0 | 0 | 0 |
| Loss of work/change in work of CP due to COVID-19 measures | 34 | 1 | 0 | 0 | 1 |
| Interruption physical and/or social activities of PWD and/or CP | 57 | 0 | 0 | 0 | 0 |
| PWD having reduced personal care | 39 | 3 | 0 | 0 | 3 |
| Increased time CP spend caring for person with dementia | 46 | 3 | 1 | 0 | 2 |
| Financial uncertainties/difficulties for PWD and/or CP | 53 | 0 | 0 | 0 | 0 |
| CP experiencing guilt over not being able to visit PWD | 22 | 0 | 0 | 0 | 0 |
| Difficulty accessing basic needs (e.g., shopping, accessing care) | 51 | 1 | 1 | 0 | 0 |
| Discontinuation/shut down of services (e.g., doctor visits) | 74 | 1 | 0 | 0 | 1 |
| Lack of access to medical support | 68 | 2 | 0 | 1 | 1 |
| Increased cost of care | 34 | 2 | 0 | 1 | 1 |
| Reduced support for PWD and/or CP | 62 | 3 | 0 | 2 | 1 |
| Increased inequities that existed before COVID-19 | 20 | 1 | 0 | 0 | 1 |
| PWD and/or CP avoiding seeking help for health issues out of fear of getting COVID-19 in hospitals or doctor surgeries | 65 | 2 | 1 | 1 | 0 |
| Lack of information on what supports and services were available for PWD and/or CP during the COVID-19 pandemic | 64 | 5 | 0 | 3 | 2 |

CP: care partner; PWD: person with dementia

**South Africa**

|  | Generic List | South Africa | | |
| --- | --- | --- | --- | --- |
|  |  | Overall | Carer | Professional |
| Diet or appetite changes of PWD and/or CP | 89 | 6 | 5 | 1 |
| Sleep disturbances for PWD and/or CP | 142 | 13 | 7 | 6 |
| Worsening physical health of PWD and/or CP | 151 | 9 | 4 | 5 |
| Faster cognitive decline of PWD | 197 | 9 | 3 | 6 |
| Increased use/dose of medications by PWD | 47 | 2 | 1 | 1 |
| PWD has difficulty understanding the COVID-19 situation | 113 | 7 | 3 | 4 |
| CP have difficulty managing COVID-19 control measures in context of PWD not understanding the situation | 85 | 8 | 5 | 3 |
| Increased anxiety/stress of PWD and/or care partners | 182 | 12 | 7 | 5 |
| PWD and/or CP feeling hopeless (e.g., feeling discouraged, loss of purpose, feeling the pandemic will never end) | 74 | 8 | 2 | 6 |
| Mood changes of PWD and/or CP (e.g., increased depression, sadness, mood swings) | 139 | 8 | 5 | 3 |
| Experiencing grief (PWD and/or CP) | 55 | 1 | 0 | 1 |
| Concerns about health of PWD | 98 | 8 | 4 | 4 |
| Concerns about health of CP | 51 | 1 | 1 | 0 |
| PWD and/or CP concerns about health of family and friends | 12 | 0 | 0 | 0 |
| CP fears of struggling to cope due to COVID-19 measures | 34 | 1 | 0 | 1 |
| PWD and/or CP concerns about the future | 46 | 0 | 0 | 0 |
| Increased behavioral problems of PWD | 111 | 8 | 5 | 3 |
| Increased social isolation of PWD and/or CP | 171 | 12 | 4 | 8 |
| Changes in quality of life and/or life satisfaction of PWD and/or CP | 83 | 1 | 0 | 1 |
| Increased care burden and/or responsibilities of CP | 119 | 7 | 4 | 3 |
| Positive/negative coping strategies used by PWD and/or CP | 32 | 3 | 0 | 3 |
| Changes in the quality of relationships for PWD and/or CP | 47 | 4 | 2 | 2 |
| Changes in social contacts of PWD and/or CP | 58 | 6 | 2 | 4 |
| Increased stigma experienced by CP during pandemic | 7 | 0 | 0 | 0 |
| Loss of work/change in work of CP due to COVID-19 measures | 34 | 3 | 1 | 2 |
| Interruption of physical and/or social activities of PWD and/or CP | 57 | 0 | 0 | 0 |
| PWD having reduced personal care | 39 | 2 | 2 | 0 |
| Increased time CP spend caring for person with dementia | 46 | 3 | 2 | 1 |
| Financial uncertainties/difficulties for PWD and/or CP | 53 | 9 | 7 | 2 |
| CP experiencing guilt over not being able to visit PWD | 22 | 0 | 0 | 0 |
| Difficulty accessing basic needs (e.g., shopping, accessing care) | 51 | 5 | 5 | 0 |
| Discontinuation/shut down of services (e.g., doctor visits) | 74 | 6 | 4 | 2 |
| Lack of access to medical support | 68 | 3 | 0 | 3 |
| Increased cost of care | 34 | 2 | 1 | 1 |
| Reduced support for PWD and/or CP | 62 | 3 | 3 | 0 |
| Increased inequities that existed before COVID-19 | 20 | 4 | 1 | 3 |
| PWD and/or CP avoiding seeking help for health issues out of fear of getting COVID-19 in hospitals or doctor surgeries | 65 | 7 | 2 | 5 |
| Lack of information on what supports and services were available for PWD and/or CP during the COVID-19 pandemic | 64 | 3 | 1 | 2 |

CP: care partner; PWD: person with dementia

**UK**

|  | Generic List | UK | | | |
| --- | --- | --- | --- | --- | --- |
|  |  | Overall | PWD | Carer | Professional |
| Diet or appetite changes of PWD and/or CP | 89 | 4 | 2 | 0 | 2 |
| Sleep disturbances for PWD and/or CP | 142 | 6 | 5 | 0 | 1 |
| Worsening physical health of PWD and/or CP | 151 | 8 | 2 | 1 | 5 |
| Faster cognitive decline of PWD | 197 | 15 | 2 | 0 | 13 |
| Increased use/dose of medications by PWD | 47 | 0 | 0 | 0 | 0 |
| PWD has difficulty understanding the COVID-19 situation | 113 | 5 | 2 | 0 | 3 |
| CP have difficulty managing COVID-19 control measures in context of PWD not understanding the situation | 85 | 7 | 1 | 1 | 5 |
| Increased anxiety/stress of PWD and/or care partners | 182 | 10 | 5 | 2 | 3 |
| PWD and/or CP feeling hopeless (e.g., feeling discouraged, loss of purpose, feeling the pandemic will never end) | 74 | 5 | 4 | 0 | 1 |
| Mood changes of PWD and/or CP (e.g., increased depression, sadness, mood swings) | 139 | 9 | 7 | 0 | 2 |
| Experiencing grief (PWD and/or CP) | 55 | 5 | 2 | 0 | 3 |
| Concerns about health of PWD | 98 | 2 | 2 | 0 | 0 |
| Concerns about health of CP | 51 | 1 | 0 | 0 | 1 |
| PWD and/or CP concerns about health of family and friends | 12 | 1 | 0 | 0 | 1 |
| CP fears of struggling to cope due to COVID-19 measures | 34 | 2 | 0 | 0 | 2 |
| PWD and/or CP concerns about the future | 46 | 1 | 1 | 0 | 0 |
| Increased behavioral problems of PWD | 111 | 10 | 3 | 0 | 7 |
| Increased social isolation of PWD and/or CP | 171 | 16 | 5 | 0 | 11 |
| Changes in quality of life and/or life satisfaction of PWD and/or CP | 83 | 5 | 2 | 0 | 3 |
| Increased care burden and/or responsibilities of CP | 119 | 4 | 1 | 0 | 3 |
| Positive/negative coping strategies used by PWD and/or CP | 32 | 0 | 0 | 0 | 0 |
| Changes in the quality of relationships for PWD and/or CP | 47 | 2 | 0 | 0 | 2 |
| Changes in social contacts of PWD and/or CP | 58 | 2 | 1 | 0 | 1 |
| Increased stigma experienced by CP during COVID-19 pandemic | 7 | 1 | 0 | 0 | 1 |
| Loss of work/change in work of CP due to COVID-19 measures | 34 | 0 | 0 | 0 | 0 |
| Interruption of physical and/or social activities of PWD and/or CP | 57 | 8 | 3 | 0 | 5 |
| PWD having reduced personal care | 39 | 1 | 0 | 0 | 1 |
| Increased time CP spend caring for person with dementia | 46 | 4 | 0 | 1 | 2 |
| Financial uncertainties/difficulties for PWD and/or CP | 53 | 1 | 1 | 0 | 0 |
| CP experiencing guilt over not being able to visit PWD | 22 | 3 | 1 | 1 | 1 |
| Difficulty accessing basic needs (e.g., shopping, accessing care) | 51 | 4 | 1 | 1 | 2 |
| Discontinuation/shut down of services (e.g., doctor visits) | 74 | 8 | 4 | 1 | 3 |
| Lack of access to medical support | 68 | 6 | 2 | 0 | 4 |
| Increased cost of care | 34 | 1 | 0 | 0 | 1 |
| Reduced support for PWD and/or CP | 62 | 4 | 1 | 0 | 3 |
| Increased inequities that existed before COVID-19 | 20 | 2 | 0 | 1 | 1 |
| PWD and/or CP avoiding seeking help for health issues out of fear of getting COVID-19 in hospitals or doctor surgeries | 65 | 8 | 3 | 1 | 4 |
| Lack of information on what supports and services were available for PWD and/or CP during the COVID-19 pandemic | 64 | 5 | 1 | 1 | 3 |

CP: care partner; PWD: person with dementia

Highlighted in yellow are the topics with the most votes within that stakeholder group
